# Supplementary material for: Prenatal exposure to polycyclic aromatic hydrocarbons and birth outcomes in a pregnancy cohort in Nairobi, Kenya
Source: PLoS One. 2026 May 13;21(5):e0347486. doi: 10.1371/journal.pone.0347486 (PMC13170848; doi:10.1371/journal.pone.0347486)
Supplement: S1 File — (PDF) [file pone.0347486.s001.pdf]

Supplementary material

Prenatal polycyclic aromatic hydrocarbon exposure and adverse birth outcomes in a pregnancy cohort in Nairobi, Kenya.

Contents

Table S1. Exposure to combustion sources indoors, outdoors, and from work-related activities 2

Table S2. Principal component loadings describing exposure to combustion sources indoors, outdoors, and from work-related activities. 3

Table S3. Characteristics of the study population by OH-PAH tertile 4

Table S4. Associations between a doubling in OH-PAH concentration and birth outcomes. 5

Table S5. Associations between the WQS Index and adverse birth outcomes constrained in the positive and negative directions. 6

Figure S1. Pearson correlation coefficients between OH-PAH metabolite 7

Figure S2. Results of sensitivity analyses describing associations between OH-PAH metabolites and birth outcomes. 8

Supplementary material
Prenatal polycyclic aromatic hydrocarbon exposure and adverse birth outcomes in a pregnancy cohort in Nairobi, Kenya.

Table S1. Exposure to combustion sources indoors, outdoors, and from work-related activities

		Response frequency (%)				
**Location**	**Exposure**	**Daily**	**Most days**	**Some days**	**Rarely**	**Never**
Indoor exposure	Incense	0	0.5	6.3	5.8	87.3
	Mosquito repellant	2.0	0.8	14.4	18.0	64.8
	Candles	0.5	0.3	1.3	81.0	17.0
	Rubbish burning	0.5	4.1	25.6	15.4	54.4
Outdoor pollutants close to the home (<20 m)	Smoke from own home	4.6	2.0	9.4	19.7	64.3
	Smoke from neighbor	5.3	25.1	15.4	12.9	41.3
Outdoor pollutants beyond the home (20m – 1km)	Cooking smoke	51.6	21.5	10.4	4.3	12.2
	Vehicle smoke	50.1	16.2	23.0	5.1	5.6
	Dumpsite	22.5	9.4	12.7	5.1	50.4
	Rubbish burning	6.6	17.5	41.5	12.4	22.0
	Welding	16.5	14.9	8.6	6.3	53.7
Exposures from work	Kerosene cooking	19.2	8.1	19.0	12.4	41.3
	Charcoal cooking	6.3	4.6	11.1	16.2	61.8
	Wood cooking	10.6	7.6	8.6	8.9	64.3
	Vehicle smoke	38.7	22.3	26.6	4.8	7.6
	Dumpsite	18.5	12.7	16.2	6.8	45.8
	Rubbish burning	5.3	15.2	40.5	14.4	24.6
	Welding	15.2	11.6	11.4	6.6	55.2
	Cigarette smoke	10.6	17.7	21.5	23.8	26.3
	Marijuana smoke	10.9	19.7	14.7	25.6	29.1

Supplementary material
Prenatal polycyclic aromatic hydrocarbon exposure and adverse birth outcomes in a pregnancy cohort in Nairobi, Kenya.

Table S2. Principal component loadings describing exposure to combustion sources indoors, outdoors, and from work-related activities.

		Principal component loading						
**Location**	**Exposure**	**PC1**	**PC2**	**PC3**	**PC4**	**PC5**	**PC6**	**PC7**
Indoors	Incense	0.07	0.10	-0.10	0.02	0.00	0.01	0.65
	Mosquito repellant	-0.01	-0.01	0.07	-0.14	0.04	-0.01	0.77
	Candles	0.14	-0.26	0.05	0.33	-0.21	0.08	0.35
	Rubbish burning	0.27	0.56	-0.16	-0.22	-0.11	0.03	-0.03
Outdoors (<20 m from home)	Smoke from own home	0.07	0.09	-0.08	0.06	0.08	0.87	0.01
	Smoke from neighbor	-0.01	0.67	-0.11	-0.15	-0.03	0.43	0.08
Outdoors (20m – 1km from home)	Cooking smoke	0.01	0.07	0.78	0.12	0.02	0.03	0.02
	Vehicle smoke	0.06	0.01	0.87	-0.02	0.12	0.04	-0.02
	Dumpsite	0.81	-0.01	0.06	-0.02	0.12	0.16	0.06
	Rubbish burning	0.74	0.25	-0.01	-0.08	-0.07	0.13	0.11
	Welding	0.12	0.06	0.07	0.03	0.83	0.17	0.03
Work-related	Kerosene cooking	0.13	-0.09	0.06	0.56	-0.32	0.20	-0.20
	Charcoal cooking	-0.03	0.06	0.05	0.79	0.20	0.05	-0.03
	Wood cooking	0.07	0.12	0.20	0.65	0.31	-0.26	0.02
	Vehicle smoke	0.08	-0.06	0.81	0.14	0.14	-0.24	-0.04
	Dumpsite	0.80	-0.07	0.10	0.22	0.18	-0.01	-0.07
	Rubbish burning	0.73	0.18	0.04	0.06	0.08	-0.20	0.04
	Welding	0.19	-0.08	0.23	0.22	0.76	-0.09	-0.06
	Cigarette smoke	0.11	0.84	0.10	0.12	0.11	0.01	-0.01
	Marijuana smoke	0.05	0.85	0.14	0.17	0.01	-0.06	0.06

Supplementary material
Prenatal polycyclic aromatic hydrocarbon exposure and adverse birth outcomes in a pregnancy cohort in Nairobi, Kenya.

Table S3. Characteristics of the study population by OH-PAH tertile

	OH-PAH tertile		
Characteristic	1 (N=118)	2 (N=118)	3 (N=117)
**Infant sex**			
Female	66 (55.9%)	58 (49.2%)	60 (51.3%)
Male	52 (44.1%)	60 (50.8%)	57 (48.7%)
**Maternal age at delivery**			
Mean (SD)	27.5 (5.82)	28.6 (5.48)	26.7 (5.34)
Median [Q1-Q3]	26.8 [22.6-32.5]	27.7 [24.0-33.1]	25.8 [23.1-30.1]
**Maternal education**			
Primary, pre-primary, or none	36 (30.5%)	26 (22.0%)	34 (29.1%)
Secondary	62 (52.5%)	76 (64.4%)	62 (53.0%)
College/University/Vocational	20 (16.9%)	16 (13.6%)	21 (17.9%)
**Maternal marital status**			
Divorced/separated/widowed	3 (2.5%)	4 (3.4%)	5 (4.3%)
Married	99 (83.9%)	106 (89.8%)	98 (83.8%)
Never married/single	16 (13.6%)	8 (6.8%)	14 (12.0%)
**Maternal employment status**			
Homemaker	66 (55.9%)	65 (55.1%)	66 (56.4%)
Employed	42 (35.6%)	49 (41.5%)	42 (35.9%)
Not employed	10 (8.5%)	4 (3.4%)	9 (7.7%)
**Parity**			
0	34 (28.8%)	18 (15.3%)	32 (27.4%)
1	30 (25.4%)	49 (41.5%)	41 (35.0%)
2	33 (28.0%)	24 (20.3%)	25 (21.4%)
3 or more	21 (17.8%)	27 (22.9%)	19 (16.2%)
**Urinary specific gravity**			
Mean (SD)	1.01 (0.00593)	1.01 (0.00649)	1.01 (0.00696)
Median [Q1-Q3]	1.02 [1.01-1.02]	1.02 [1.01-1.02]	1.02 [1.01-1.02]
**Urinary cotinine**			
< 10 ng/mL	116 (98.3%)	113 (95.8%)	112 (95.7%)
10-200 ng/mL	1 (0.8%)	3 (2.5%)	3 (2.5%)
>200 ng/mL	1 (0.8%)	2 (1.7%)	2 (1.7%)
**Dirty fuel use**			
Daily or most days	18 (15.3%)	37 (31.4%)	64 (54.7%)
Some days	37 (31.4%)	32 (27.1%)	24 (20.5%)
Rarely or not at all	63 (53.4%)	49 (41.5%)	29 (24.8%)

Supplementary material
Prenatal polycyclic aromatic hydrocarbon exposure and adverse birth outcomes in a pregnancy cohort in Nairobi, Kenya.

Table S4. Associations between a doubling in OH-PAH concentration and birth outcomes.

		Coefficient and 95% CI	
**Outcome**	**OH-PAH**	**Primary Model**	**Co-exposure Model**
Birth weight (kg)	2-OH-NAP	0.022 (-0.024, 0.068)	0.028 (-0.021, 0.076)
	2/9-OH-FLUO	0.010 (-0.036, 0.056)	0.011 (-0.038, 0.060)
	2-OH-PHEN	0.006 (-0.047, 0.058)	0.005 (-0.050, 0.060)
	3-OH-PHEN	0.025 (-0.024, 0.073)	0.024 (-0.027, 0.076)
	1-OH-PYR	0.038 (-0.011, 0.087)	0.045 (-0.008, 0.097)
Gestational age (days)	2-OH-NAP	-0.518 (-1.821, 0.786)	0.040 (-1.332, 1.412)
	2/9-OH-FLUO	-0.784 (-2.087, 0.518)	-0.351 (-1.729, 1.028)
	2-OH-PHEN	-1.606 (-3.092, -0.120)	-1.309 (-2.862, 0.243)
	3-OH-PHEN	-0.108 (-1.495, 1.279)	0.295 (-1.160, 1.749)
	1-OH-PYR	-0.629 (-2.032, 0.773)	-0.281 (-1.771, 1.209)
BW-GA Z-score	2-OH-NAP	0.064 (-0.054, 0.181)	0.047 (-0.077, 0.171)
	2/9-OH-FLUO	0.058 (-0.055, 0.171)	0.038 (-0.082, 0.158)
	2-OH-PHEN	0.079 (-0.053, 0.210)	0.061 (-0.076, 0.198)
	3-OH-PHEN	0.044 (-0.079, 0.167)	0.021 (-0.108, 0.150)
	1-OH-PYR	0.112 (-0.009, 0.232)	0.110 (-0.018, 0.239)
Preterm birth	2-OH-NAP	0.966 (0.698, 1.337)	0.844 (0.576, 1.237)
	2/9-OH-FLUO	1.288 (0.882, 1.881)	1.184 (0.772, 1.816)
	2-OH-PHEN	1.496 (0.940, 2.381)	1.429 (0.889, 2.298)
	3-OH-PHEN	0.996 (0.678, 1.465)	0.908 (0.567, 1.455)
	1-OH-PYR	0.989 (0.701, 1.395)	0.905 (0.604, 1.358)

Supplementary material
Prenatal polycyclic aromatic hydrocarbon exposure and adverse birth outcomes in a pregnancy cohort in Nairobi, Kenya.

Table S5. Associations between the WQS Index and adverse birth outcomes constrained in the positive and negative directions.

Outcome	Direction	Primary model	Co-exposure model
Birth weight (kg)	Negative	-0.004 (-0.023, 0.016)	-0.005 (-0.025, 0.016)
	Positive	0.004 (-0.017, 0.024)	0.005 (-0.018, 0.027)
Gestational age (days)	Negative	-0.44 (-0.98, 0.10)	-0.32 (-0.88, 0.23)
	Positive	0.06 (-0.46, 0.58)	0.25 (-0.34, 0.83)

Supplementary material

Prenatal polycyclic aromatic hydrocarbon exposure and adverse birth outcomes in a pregnancy cohort in Nairobi, Kenya.

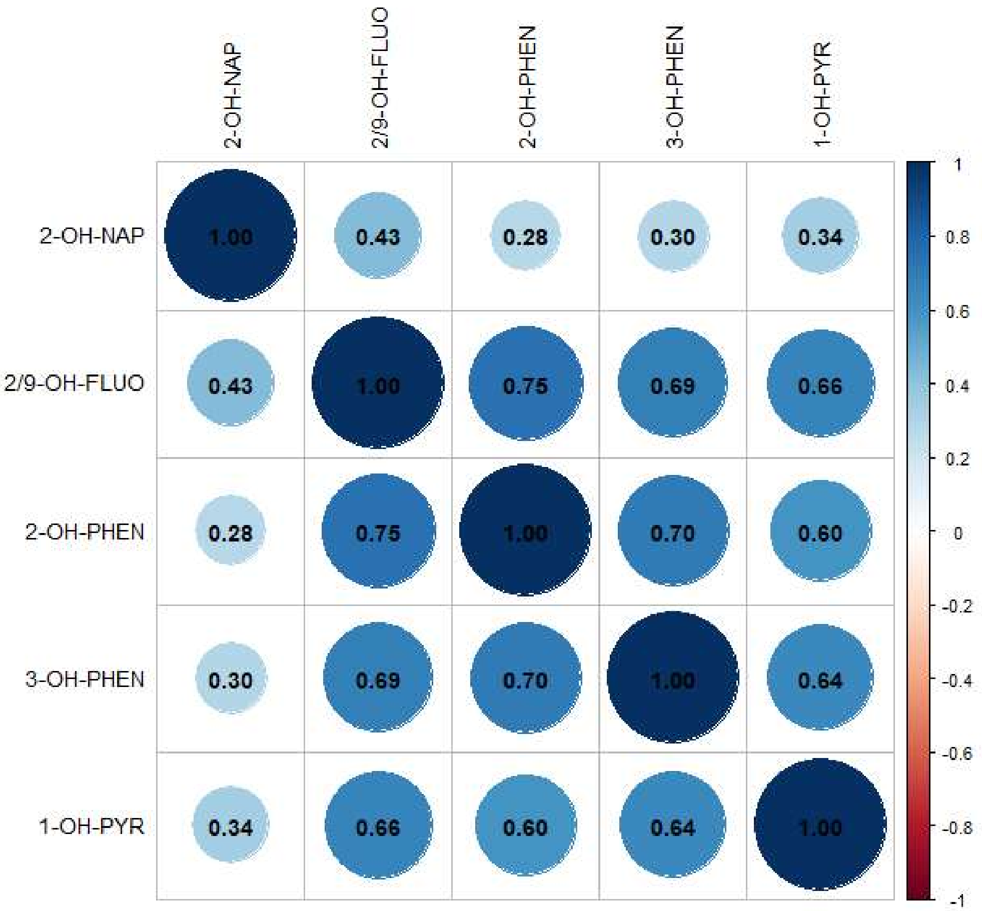

Figure S1. Pearson correlation coefficients between OH-PAH metabolite

Supplementary material
Prenatal polycyclic aromatic hydrocarbon exposure and adverse birth outcomes in a pregnancy cohort in Nairobi, Kenya.

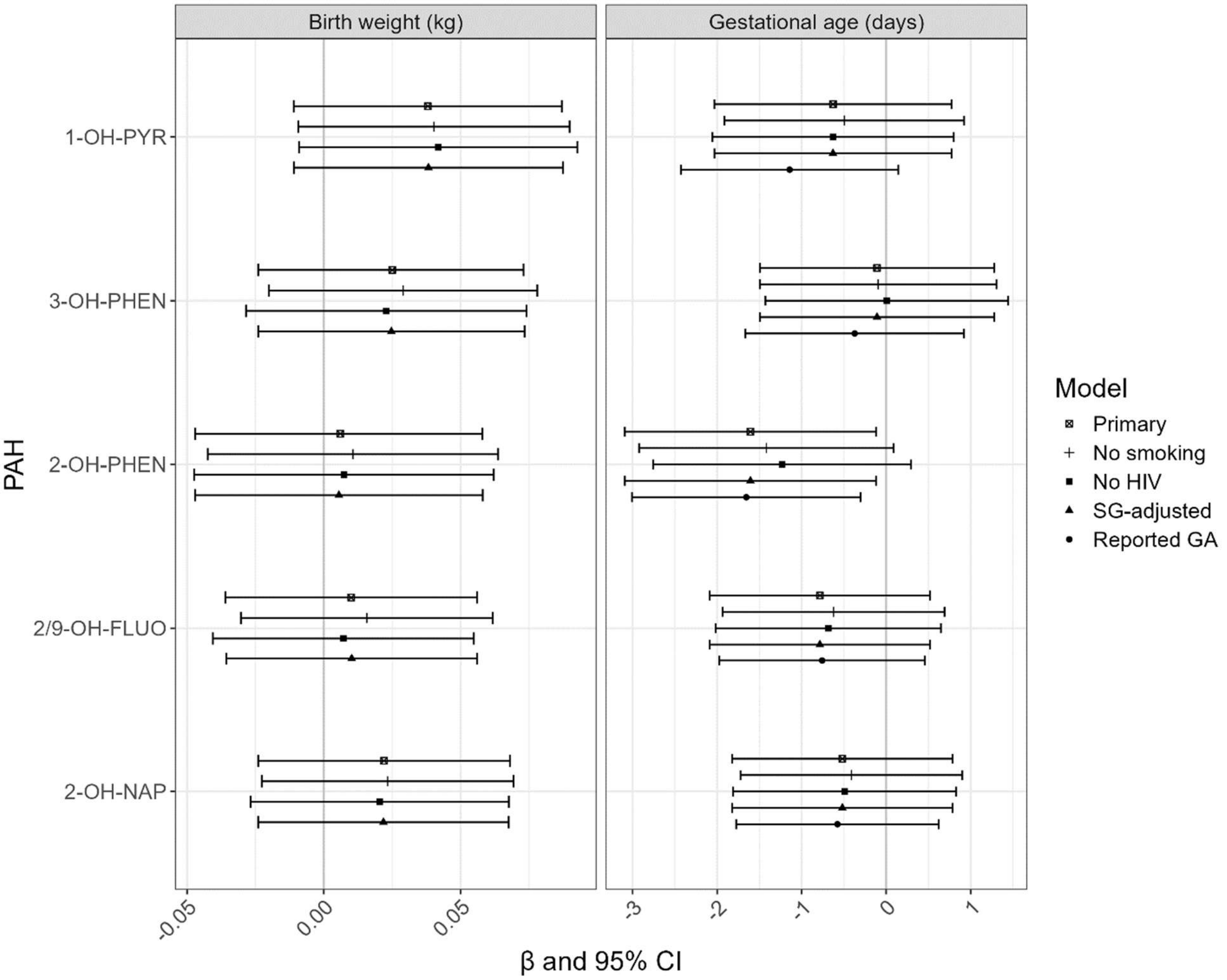

Figure S2. Results of sensitivity analyses describing associations between OH-PAH metabolites and birth outcomes.
